# The Relation Between Goal‐Predictive Gaze Behavior and Imitation—A Live Eye‐Tracking Study in 12‐Month‐Olds

**DOI:** 10.1111/infa.70050

**Published:** 2025-11-04

**Authors:** Franziska Sieber, Jan Czarnomski, Moritz M. Daum, Norbert Zmyj

**Affiliations:** ^1^ TU Dortmund University Dortmund Germany; ^2^ University of Zurich Zurich Switzerland

**Keywords:** cognitive development, goal‐predictive gaze shifts, imitation, infants, live eye‐tracking

## Abstract

Children learn from others by imitating observed behavior. According to some theorists, to imitate an agent's action, infants need to identify the agent's action goal. To test this assumption, goal‐predictive gaze shifts of 104 German 12‐month‐olds (57 female) were measured using live eye‐tracking. These goal‐predictive gaze shifts were related to their imitation of an action performed by a live model. This relationship was controlled for in terms of cognitive developmental status. We used one task of the imitation battery FIT 12 and analyzed the infants' imitation and goal‐predictive gaze shifts. The infants showed goal‐predictive gaze shifts to actions presented at a realistic speed. Furthermore, imitation was related to their goal‐predictive gaze shifts. This association was partially explained by cognitive‐developmental status, which should be considered an important factor in the development of imitation.

## 1 Introduction

Imitation is central to early human development, as it allows for the transmission of cultural practices and knowledge (Tomasello et al. 2005). The development of imitation in childhood and the factors that influence it, such as situational, model, or task characteristics, have been widely studied over the past decades (e.g., Barr et al. 1996; Wood et al. 2013; Zmyj and Seehagen 2013). However, the mechanisms underlying imitation and its development still require further investigation. Defining imitation as encompassing an understanding of the model's intention implies that to imitate, infants need to identify and understand the action goals of others (e.g., Carpenter and Call 2009). One possibility for assessing goal understanding in infants is to measure their goal‐predictive gaze behavior (see Hunnius and Bekkering 2014), which should be related to imitation if goal understanding is indeed one of the mechanisms underlying imitation. The present study, therefore, aimed to investigate the relation between imitation and goal‐predictive gaze shifts by testing 12‐month‐olds in a live eye‐tracking setting. This setting further allowed for the investigation of goal‐predictive gaze shifts in a more naturalistic context, with a live model presenting actions at a realistic speed.

### 1.1 Imitation

Studies investigating imitation are initially faced with the challenge of defining imitation itself. Depending on the main research question, most definitions can be assigned to one of two main categories: Broader, behavior‐based definitions conceive imitation as copying any observed behavior (e.g., Barr et al. 1996; Paulus 2011), whereas narrow, intention‐based definitions specify imitation as copying the means of an action to achieve a specific goal (e.g., Carpenter and Call 2009; Tomasello et al. 2005). Depending on the definition employed, different potentially relevant mechanisms underlying imitation may be suggested. According to behavior‐based definitions, attention to the demonstrated action is the first part of the imitative process (Bandura 1971). However, studies with infants in the first 2 years of life have shown that the duration of looking at an observed action is not correlated with the imitation of this action (Kolling et al. 2014; Óturai et al. 2013; Sonne et al. 2016).

Intention‐based definitions emphasize the role of means and goals in imitation (e.g., Gergely et al. 2002; Tomasello et al. 2005). Imitation includes those behaviors in which an infant reproduces the goal of the model and the specific means that have led to this goal. Actions are construed as movements performed by an agent to elicit a desired goal. Accordingly, actions are represented in terms of their goals (Prinz 1997), and infants must identify the goal of an action in order to be able to imitate it (Tomasello et al. 2005). Thus, according to these approaches, imitation expresses what is typically called “infants' action understanding” (Gampe et al. 2016).

### 1.2 Goal Prediction

The question of how young children perceive the behavior of others is central to developmental psychology (Hunnius and Bekkering 2014), and infants' action understanding plays a key role in this field of research. Recent theoretical accounts of infants' goal perception emphasize that there is not a single pathway to action understanding. Contemporary frameworks often distinguish between experience‐based and cue‐based theories (e.g., Bíró and Leslie 2007; Gredebäck and Falck‐Ytter 2015). Experience‐based accounts propose that infants' ability to map and predict action goals is rooted in their own motor experience and shared action–perception representations (e.g., Kanakogi and Itakura 2011; Meltzoff 2005). In contrast, cue‐based approaches argue that infants can also interpret unfamiliar actions as goal‐directed by relying on observable agency cues, such as self‐propelled movement, equifinality, salient action effects, or spatial information (e.g., Bíró and Leslie 2007; Gönül et al. 2024; Southgate 2013). This more nuanced view highlights that predictive gaze shifts in infancy can be supported by both prior motor experience and by contextual cues, offering a broader framework for interpreting early goal prediction.

Empirical research has frequently relied on infants' eye movements to capture how they anticipate others' actions (e.g., Elsner and Adam 2021; Ganglmayer et al. 2019; Gönül et al. 2024). One prominent way to assess infants' action understanding is to capture their goal prediction through goal‐predictive gaze shifts during an ongoing action using eye‐tracking (Falck‐Ytter et al. 2006). Goal prediction refers to the ability to anticipate the goal of an observed action before the action is completed, and goal‐predictive gaze shifts have been used to measure action understanding both in adults (e.g., Flanagan and Johansson 2003) and infants (e.g., Falck‐Ytter et al. 2006; Melzer et al. 2012). Infants demonstrate goal‐predictive gaze shifts from early in life, as measured through anticipatory looking at screen‐based events. For instance, even 6‐month‐olds displayed predictive looks to familiar actions (i.e., bringing a cup to one's mouth or a phone to one's ear; Hunnius and Bekkering 2010).

### 1.3 Relation Between Imitation and Goal Prediction

The two conceptual accounts of intention‐based imitation and goal prediction can be conceived as depicting infants' action understanding. However, to the best of our knowledge, only one previous study has investigated the relation between imitation and goal‐predictive gaze shifts. Gampe et al. (2016) presented 12‐, 18‐, 24‐, and 30‐month‐olds with two multi‐step actions on a computer screen: one familiar action (i.e., hammering) and one unfamiliar action (i.e., pulling) for this specific context (i.e., inserting a building block in a box). The children's gaze behavior was analyzed during the demonstration. Each action condition (hammering and pulling) was divided into four action sequences of four steps each. For each action step in both conditions, infants' gaze latency was calculated, that is, the difference between the time that the hand/tool/object entered the AOI of the respective action step and the time of the infants' first fixation within that same AOI. After the demonstration, the children received the objects, and their imitation of these actions was measured. The faster infants predicted the goal in the familiar condition, the more likely they were to imitate the observed action, while this was not the case in the unfamiliar condition. These results suggest that goal‐predictive gaze shifts are related to imitation of familiar actions but not unfamiliar actions.

Two criticisms can be raised regarding the methodology used by Gampe et al. (2016). First, the demonstrated actions differed from a lifelike presentation in several ways. The actions were presented on a digital screen, which could have underestimated young children's performance due to the video deficit effect, according to which infants show a lower ability to transfer learning from videos compared to learning from equivalent live experiences (Anderson and Pempek 2005). Analogously to other related studies, the demonstration video showed only the main elements of the action and no distractors (e.g., Falck‐Ytter et al. 2006; Hunnius and Bekkering 2010; Melzer et al. 2012) and was presented at a relatively slow speed (i.e., 1000–1960 ms per action step, see also Ambrosini et al. 2013). Action duration plays an important role in infants' goal‐predictive gaze shifts, with research showing that slower action demonstrations result in more goal‐predictive gaze shifts in 12‐month‐olds (Daum et al. 2016).

Nevertheless, slower action demonstrations do not reflect the everyday learning environment of young children. Monroy et al. (2021) were the first to investigate action prediction in a free‐flowing real‐life setting during infant‐parent interactions and reported that 9‐month‐olds anticipated their parents' goal‐directed movements. Another study by Rosander and von Hofsten (2011) found goal‐predictive gaze shifts in 11‐month‐olds in a live interaction with the experimenter for actions performed by themselves as well as actions performed by the experimenter using electrooculography (EOG). Beyond that, little is known about whether infants can predict another's action goals in live interactions. Second, Gampe et al. (2016) did not control for children's general cognitive development, for example using the Bayley Scales of Infant and Toddler Development III (BSID‐III, Bayley 2006). Accordingly, imitation and anticipatory looking might result from changes in cognitive development rather than the prediction of goals.

### 1.4 The Present Study

The aim of the present study was twofold. First, we aimed to investigate infants' goal‐predictive gaze shifts in a more naturalistic context via live eye‐tracking, with a live model presenting the action. Second, we aimed to investigate the relation between goal‐predictive gaze shifts and imitation of goal‐directed actions while controlling for infants' cognitive developmental status. Therefore, creating a novel approach, we investigated 12‐month‐olds’ goal‐predictive gaze shifts during action observation in a live eye‐tracking setting and examined their association with their imitation of the observed action, and additionally employed the Cognitive Scale of the BSID‐III (Bayley 2006) in order to control for infants' cognitive development.

First, we hypothesized that infants would show goal‐predictive gaze shifts in live settings using an action with realistic speed. Second, we hypothesized that infants who imitated a goal‐directed action would show faster goal‐predictive gaze shifts during the demonstration of this action than infants who did not imitate. Third, we hypothesized that this association between imitation and goal‐predictive gaze shifts would remain constant after controlling for infants' cognitive development.

## 2 Method

### 2.1 Participants

The participating families were recruited as part of a longitudinal study from a database of families who had previously signed up to participate in child development studies. The families lived in one of two medium‐sized cities in northwestern Germany. The final sample comprised 104 twelve‐month‐olds (57 girls; *M* = 360 days, SD = 9; range 344–386). Fifty‐two additional children had to be excluded because they did not complete the test phase of the FIT (*n* = 2), had less than two valid eye‐tracking trials (*n* = 15), did not complete the test phase of the FIT, and had less than two valid eye‐tracking trials (*n* = 2), or due to technical problems during calibration of the eye‐tracking system (*n* = 33). All of the children were White. The majority of the parents had either a university degree (mothers 61%, fathers 51%) or a university entrance‐level diploma (mothers 18%, fathers 16%) as their highest educational attainment. Parents provided informed consent for their children to participate in the study. Children received a small gift, and parents received 10 Euros after the session. The present study was approved by the local Ethics Committee of the Faculty of Psychology at XXX. The research was conducted in accordance with APA ethical standards in the treatment of the study sample.

An a priori G*power 3.1 analysis was run to determine the appropriate sample size for the longitudinal study, which showed that a sample of 85 participants was sufficient to achieve 80% power and a medium effect size (Faul et al. 2007). To ensure we get data from 85 participants at each test point, we invited 160 children and their families. In addition, a post hoc G*Power 3.1 analysis was performed to determine the statistical power of the current study and detect an effect of goal prediction on imitation. Given the final sample size of *N* = 104, the calculated effect size *f* ^2^ = 0.074 with an alpha level set at 0.05 and a total of two predictors, the power achieved was 79%.

### 2.2 Apparatus and Stimuli

**FIGURE 1:**
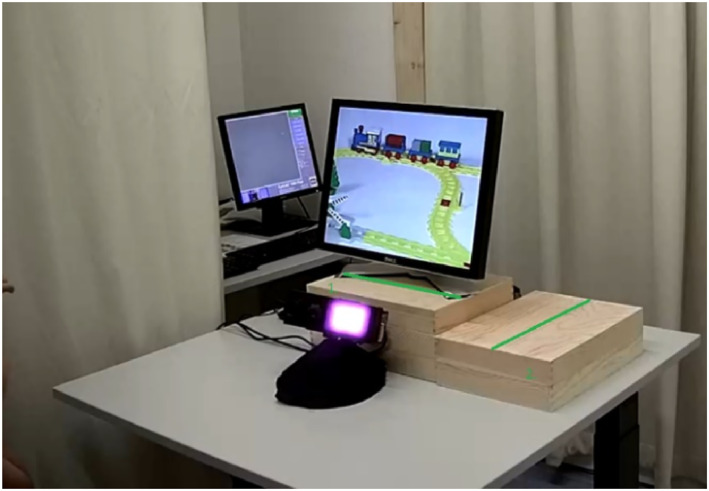
Experimental setup during calibration.

**FIGURE 2:**
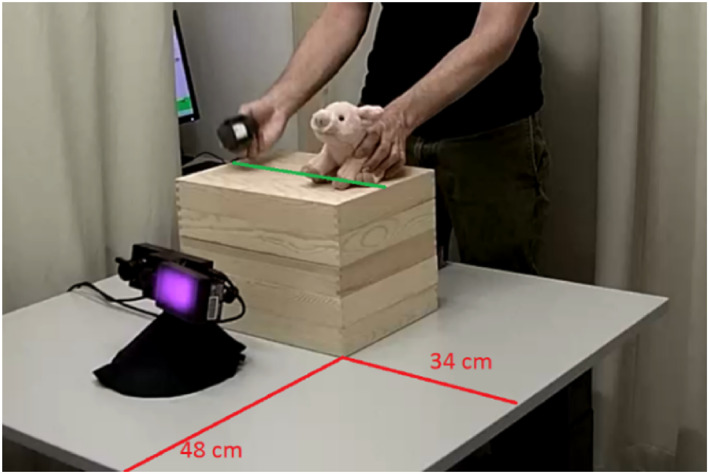
Experimental setup during demonstration.

Gaze behavior was recorded using an EyeLink 1000 Plus eye tracker (SR Research, Ottawa, Canada). During calibration, the eye tracker was mounted on a 17‐inch screen with a resolution of 1280 × 1024 pixels. The sampling rate was 500 Hz. During the eye‐tracking session, infants sat on their caregiver's lap, facing the experimenter at the opposite side of the table, approximately 60 cm away from the eye tracker. The calibration screen was placed on the table on a wooden box (27 × 15 × 36 cm; see Figure 1). When the screen was removed, another wooden box (27 × 10 × 36 cm) was placed on top of the first (see Figure 2).

The material used in the imitation task was as close as possible to the material used in the original task from the FIT 12 (Goertz et al. 2006). Infants were presented with a pink stuffed toy pig (height 16 cm) wearing a brown hat attached with Velcro.

### 2.3 Experimental Setup and Procedure

To test infants' imitation, we used the Frankfurt Imitation Test for 12‐month‐olds (FIT 12, Goertz et al. 2006). The FIT 12 entails five tasks presented in a fixed order: *Tin can*, *Pig*, *Cup & knife*, *Mouse*, and *Drum*. The infants were presented with all five tasks. For the present study, we only used the *Pig* task because it is the only task that enabled us to measure anticipatory looks during action observation. The other tasks either do not entail target actions that could be used to capture goal prediction (Tin can, Cup & knife, Mouse) or cannot be analyzed due to spatial overlap of the action steps (Drum; see https://osf.io/7rhzk/?view_only=8ac6570a6cf146819410c5803f4faa24 for one FIT demonstration phase containing all tasks).

For the purpose of the present study, we adapted the original version of the Pig task in two ways: First, we implemented a baseline phase to assess whether the infants spontaneously showed the target behavior (Zmyj et al. 2017). Second, we awarded an extra point because we conducted a two‐step action compared to the original single step. We added the Action Step 2 “putting the hat back on the pig” to the original Action Step 1 “removing the hat from the head of the pig” in order to assess goal‐predictive gaze shifts (see also Óturai et al. 2012; Zmyj et al. 2017).

After the child and their caregiver arrived at the laboratory, the experimental session began with a short warm‐up phase, in which the experimenter played with the infants until they felt comfortable, that is, until the child smiled at him and gave him a toy back during play. Next, the study started with the baseline phase of the FIT. Infants were seated in a highchair facing the experimenter at the opposite side of the table. The experimenter gave the infant one object after another for 30 s each, without any prior demonstration of the target action. The hat was placed on the pig's head when the experimenter handed the object to the infants. For the subsequent demonstration phase, the infants were moved to another table in the same room, where the eye tracker was located. Eye‐tracking started with a five‐point calibration procedure, during which a small yellow duck expanded and contracted on the screen. The calibration screen was placed on a wooden box, and the eye tracker was placed in front of this box (see Figure 1). After calibration, the experimenter removed the screen and positioned another wooden box onto the first wooden box. Subsequently, the experimenter started the demonstration phase, in which he demonstrated the action live on the wooden boxes on a marked line so that the demonstration height corresponded to the center of the calibration (see Figure 2). The experimenter removed the hat from the pig's head and then put the hat back on. This sequence was presented four times. The action demonstration can be divided into *two steps*: taking the hat off the pig's head (Action Step 1) and putting the hat back on (Action Step 2; see https://osf.io/7rhzk/?view_only=8ac6570a6cf146819410c5803f4faa24 for an example of a demonstration phase of the FIT). As only Action Step 2 allows the measurement of goal‐predictive gaze shifts, we excluded Action Step 1 from the analyses. Action Step 2 (putting the hat back on) can be further divided into *three phases*: the hat resting motionless at the starting point of Action Step 2 until it begins to move toward the pig (Phase 1), the moving phase during which the hat moves toward the pig's head until it enters the Goal Area of Interest (AOI; Phase 2), and the phase when the hat enters the Goal AOI and is placed on the pig's head until it is removed again (Phase 3). The mean total durations of Action Step 2 (“putting the hat back on”), the three phases and the anticipation period, the time that he infants had to anticipate the action before the goal was reached, are presented in Table 1.

**TABLE 1:** Mean duration of the three phases of action step 2, mean total duration of action step 2 and mean duration of the anticipation period within step 2 with standard deviation in parentheses, minimum and maximum all in ms.

	*M* (SD)	Min	Max
Action step 2 “Putting hat back on”	3331 (357)	2214	4189
Phase 1 “Hat motionless”	1433 (310)	352	2280
Phase 2 “Hat moving toward goal”	608 (92)	324	933
Phase 3 “Hat placed on pig's vertex”	1289 (181)	887	1880
Anticipation period sum of Phase 1 and Phase 2	2013 (369)	893	2842

*Note:* The anticipation period covers the time the infant had to anticipate the action from the start of Phase 1 to the end of Phase 2.

After the demonstration phase, infants returned to the first table. Due to time constraints, we reduced the delay between the demonstration and test phase from 30 min in the original version of the FIT (Goertz et al. 2006) to 15 min. During the 15‐min delay, two tasks assessing infants' temperament were conducted (as part of the longitudinal project), and a break of 5 min was included. The temperament data are not reported here because they address theoretically different questions. After the delay, the FIT test phase started. Infants received the objects again and could play with them for 30 s each. Again, the hat was placed on the pig's head when the experimenter handed the object to the infants. The session ended with assessing infants' cognitive developmental status using the Cognitive Scale of the BSID‐III, following the guidelines in the manual (Bayley 2006). The Cognitive Scale provides standardized scores for cognitive‐developmental status (*M* = 100, SD = 15). The whole session lasted for approximately 60 min and was video‐recorded.

### 2.4 Data Processing

#### 2.4.1 Eye‐Tracking Data

**FIGURE 3:**
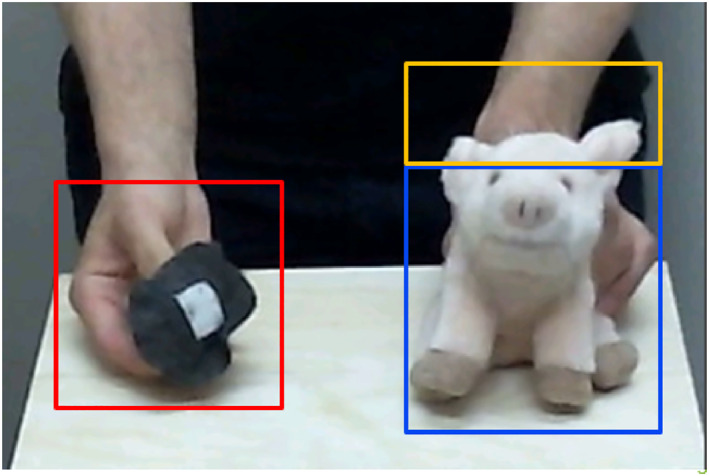
Exemplary definition of the AOIs for One Trial. Red = dynamic Hat AOI; yellow = static Goal AOI; blue = static Pig AOI.

At the time when the present study was conducted, there was no available ready‐to‐use set‐up for live eye tracking. Thus, the videos were initially synchronized offline by hand in order to align the eye‐tracking data with the video files using ffmpeg (https://ffmpeg.org). To analyze goal‐predictive gaze shifts, we defined three areas of interest (AOIs) covering the hat (dynamic Hat AOI), the vertex of the pig's head as the intended target (static Goal AOI), and the pig's body, including its face and eyes, starting from below the vertex (static Pig AOI) for each trial (see Figure 3). The dynamic Hat AOI was added frame by frame to match the gaze trace with the moving hat. Given that the action was demonstrated live and was not screen‐based, the AOIs were defined manually for each child in each demonstration trial. Therefore, the size varied minimally between the trials (see Supporting Information S1 for definition criteria of the AOIs). We included the Pig AOI because infants show a preference for eyes in faces as the most salient cue (Wagner et al. 2013). To avoid falsely including gaze shifts on the pig's eyes or the nose as goal‐predictive gaze shifts, we added this third AOI covering the pig's face and body, separate from the relevant goal AOI covering the vertex of the pig's head. A trial was considered valid if infants met the following criteria: First, they had to fixate the Hat AOI, which covers the hat and parts of the demonstrator's hand, for at least 100 ms before shifting their gaze to the future location of the hat, the Goal AOI, which covers the pig's head. Second, positive gaze‐arrival times had to be less than 2000 ms to be included as a valid trial, with gaze‐arrival times over 2000 ms excluded as outliers. Infants had to complete at least two valid trials to be included in the analysis. We did not employ the conservative criterion that gaze shifts are predictive only if the gaze is shifted after the hat starts to move (e.g., Falck‐Ytter et al. 2006). Instead, we chose a more liberal criterion, including gaze shifts, even if they appeared before the start of the movement. Unlike many other studies on goal‐predictive gaze shifts, infants could see the experimenter's motionless hand after he had removed the hat and could, therefore, perform goal‐predictive gaze shifts before the movement had started (for a similar approach, see Henrichs et al. 2012, 2014).

We also included the first trial in the analysis (see also Cannon et al. 2012) because infants had seen the action goal (the hat on the pig's head) twice before, in the baseline phase and at the beginning of the demonstration.

Gaze‐arrival times were calculated by subtracting the time infants first fixated on the Goal AOI from when the hat entered the Goal AOI for each trial. Thus, positive gaze‐arrival times represent goal‐predictive gaze shifts, whereas negative gaze‐arrival times represent reactive gaze shifts. Mean gaze‐arrival times were calculated for each infant using an average across all valid trials.

Two observers each coded half of the videos independently using the software Data Viewer (SR Research, Ottawa, Canada). To assess interrater reliability, a third independent observer coded a random selection of 41 videos (37%), approximately half of which were coded by Coder 1 and half by Coder 2. Interrater reliability for the mean gaze‐arrival time was excellent, at *r* = 0.893, *F* (40, 40) = 11.010, *p* < 0.001, Intraclass Correlation Coefficient (ICC (2, 1, absolute agreement).

#### 2.4.2 Imitation

Infants' imitation was coded from the videos by one observer. Infants were rated as “imitators” if they put the hat back on the pig's head in the test phase or if the hat made contact with the pig's head, indicating that they attempted to do so. They were rated as “non‐imitators” if they did not reproduce Action Step 2. Another independent observer additionally coded the behavior of 60 randomly chosen infants. The interrater reliability for imitation was excellent, at *r* = 0.924, *F* (59, 59) = 13.632, *p* < 0.001, ICC (2, 1, absolute agreement).

#### 2.4.3 Cognitive Scale (BSID‐III)

The coding of the Cognitive Scale of the BSID‐III followed the guidelines in the technical manual (Bayley 2006). An independent second observer additionally coded the behavior of 59 randomly chosen infants. Interrater reliability for the BSID‐III scores was excellent, *r* > 0.958, *F* (58, 58) = 24.832, *p* < 0.001, ICC (2, 1, absolute agreement).

## 3 Results

In the baseline phase of the FIT, no infant showed Action Step 2 “putting the hat back on”. In the test phase of the FIT, 18 infants (17%) imitated Action Step 2. The mean score on the Cognitive Scale (BSID‐III) was 79 (SD = 15). Infants' mean gaze‐arrival time at the static Goal AOI was *M* = 288 ms (SD = 464). The one‐sample *t*‐test against the threshold of 0 ms revealed a significant effect, *t* (103) = 6.313, *p* < 0.001, *d* = 0.62, indicating that, on average, infants showed goal‐predictive gaze shifts.

We first tested whether imitators (*n* = 18) were generally more attentive to the action demonstration than non‐imitators (*n* = 86) by comparing the total looking time at all three AOIs, averaged over all four trials, of imitators and non‐imitators for Action Step 2. An independent samples *t*‐test revealed no significant difference in the mean total looking time between imitators (*M* = 2679 ms, SD = 531) and non‐imitators, *M* = 2468 ms, SD = 1049; *t* (102) = −0.827, *p* = 0.410, *d* = 0.22.

**FIGURE 4:**
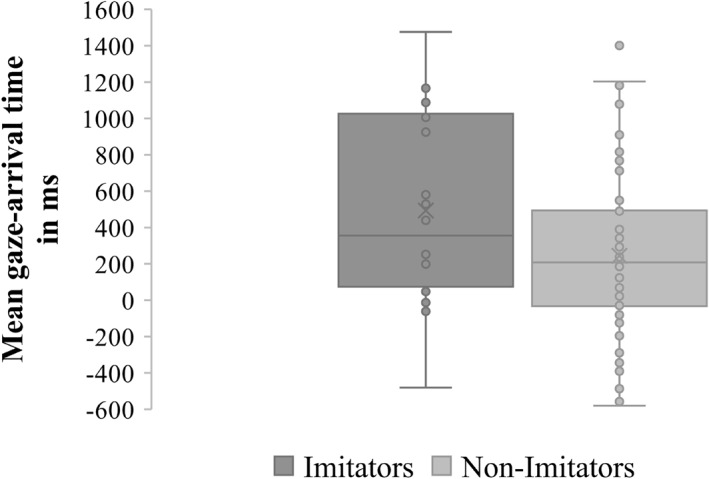
Box plots representing mean gaze‐arrival times at the goal AOI of imitators and non‐imitators. Circles represent the mean gaze‐arrival time of every infant across all four trials. Crosses represent the mean gaze‐arrival times of the imitators and non‐imitators. Imitators: *n* = 18; non‐imitators: *n* = 86.

The mean number of valid trials, the mean score on the Cognitive Scale (BSID‐III), and the mean gaze‐arrival times for all four trials, all compared separately for imitators and non‐imitators, are shown in Table 2. We then compared the mean gaze‐arrival times across all four trials of imitators (*M* = 493, SD = 530) and non‐imitators (*M* = 245, SD = 441; see Figure 4). One‐sample *t*‐tests against the threshold of 0 ms revealed significant effects for imitators, *t* (17) = 3.947, *p* < 0.001, *d* = 0.93, and non‐imitators, *t* (85) = 5.143, *p* < 0.001, *d* = 0.555, indicating that both imitators and non‐imitators show goal‐predictive gaze shifts. Comparing the mean gaze‐arrival times of imitators and non‐imitators, an independent samples *t*‐test revealed that imitators showed significantly faster goal‐predictive gazes than did non‐imitators, *t* (102) = −2.098, *p* = 0.038, *d* = 0.40.

**TABLE 2:** Mean number of valid trials, mean score of BSID‐III, and mean gaze‐arrival times (GAT) of all four trials, compared separately for imitators and non‐imitators of action step 2 including results of group comparisons.

	Imitators	Non‐imitators	*t*	*df*	*p*	Cohen's *d*
	*M* (SD)	*M* (SD)				
Number of valid trials^a^	3.67 (0.77)	3.35 (0.71)	−1.693	102	0.094	0.44
Cognitive scale (BSID‐III)^b^	87 (16)	77 (13)	−2.723	101	0.008	0.74
GAT Trial 1^c^	651 (754)	162 (565)	−2.526	20	0.020	0.81
GAT Trial 2^c^	564 (778)	413 (619)	−0.862	92	0.391	0.23
GAT Trial 3^c^	399 (780)	196 (766)	−0.947	81	0.346	0.26
GAT Trial 4^c^	372 (811)	240 (636)	−0.700	77	0.486	0.20

*Note:* Imitators: *n* = 18; non‐imitators: *n* = 86. The size of the two groups varies for the gaze‐arrival times of the four trials depending on whether the trials were valid for the respective participant.

^a^ Scores between 2 and 4 are possible.

^b^ Scores of the BSID‐III are standardized with *M* = 100, SD = 15.

^c^ *M* and SD are presented in ms.

Next, we conducted a hierarchical logistic regression (inclusion method) with the FIT 12 test score, defined as the imitation score that was coded as 0 (Action step 2 not shown) or 1 (Action step 2 shown), as dependent variable. In the first step, we added infants' cognitive developmental status (BSID‐III score) as control variable. In the second step, we added infants' mean gaze‐arrival time as a predictor. The final model of hierarchical logistic regression model was significant, *χ* ^*2*^ (2) = 8.816, *p* < 0.012, Nagelkerke's *R* ^2^ = 0.136. The cognitive developmental status predicted the imitation score (*p* = 0.035), increasing the likelihood of imitating Action step 2, OR = 1.042, whereas the mean gaze‐arrival time did not uniquely contribute to the model (*p* = 0.172). All model coefficients are depicted in Table 3.

**TABLE 3:** Hierarchical logistic regression analysis predicting the imitation score (yes/no).

Model and predictors	Model				*B*	Wald	*p*	OR	95% CI for OR
	*χ* ^2^	*df*	*p*	Nag *R* ^2^					
Model 1	6.948**	1	0.008	0.108					
Constant					−5.465	11.423	< 0.001	0.004	
Cognitive scale (BSID‐III)					0.048*	6.451	0.011	1.049	[1.011, 1.089]
Model 2	8.816*	2	0.012	0.136					
Constant					−5.209	10.165	0.001	0.005	
Cognitive scale (BSID‐III)					0.041*	4.459	0.035	1.042	[1.003, 1.083]
Mean gaze‐arrival time					0.001	1.867	0.172	1.001	[1.000, 1.002]

Abbreviations: CI = confidence interval; OR = odds ratio; Nag *R* ^2^ = Nagelkerke *R* ^2^.

*p < 0.05.

**p < 0.01.

Since the duration of the individual phases of the live demonstration varied, as did the amount of time infants had to anticipate the action (see Table 1), we additionally calculated a correlation between the mean anticipation period and mean gaze‐arrival time. The longer the anticipation period, the higher the gaze‐arrival times, that is, the faster the infants anticipated, *r* = 0.41, *p* < 0.001.

Given the restrictive nature of the imitation task, that is, infants could only imitate Action Step 2 (“putting the hat back on”) if they imitated Action Step 1 (“removing the hat”), we conducted some additional analyses. First, we calculated the percentage of infants who imitated Action Step 2 based on the number of infants who imitated Action Step 1, resulting in an imitation rate of 29%. Second, we focused on the non‐imitators in Action Step 2. Of the *n* = 86 infants who did not imitate Action Step 2, 44 (51%) had imitated Action Step 1, whereas 42 (49%) had not imitated Action Step 1. Their Cognitive Scale scores and mean gaze‐arrival times are presented in Table 4. Comparing the mean gaze‐arrival times of these Step 1 imitators (*n* = 44) and non‐imitators (*n* = 42), an independent samples *t*‐test revealed no significant differences, *t* (84) = 2.362, *p* = 0.131, *d* = −33.

**TABLE 4:** Mean score of BSID‐III, and mean gaze‐arrival times (GAT) of non‐imitators of action step 2 based on their imitation of action step 1 (“Removing the Hat”) including results of group comparisons.

	Action step 1 imitators	Action step 1 non‐imitators	*t* (84)	*p*	Cohen's *d*
	*M* (SD)	*M* (SD)			
Cognitive scale (BSID‐III)	78 (14)	76 (13)	−0.519	0.605	0.15
Mean gaze‐arrival time^a^	174 (389)	318 (484)	2.362	0.131	−0.33

*Note:* Action Step 1 imitators: *n* = 44; Action Step 1 non‐imitators: *n* = 42.

^a^ *M* and SD are presented in ms.

## 4 Discussion

The present study investigated the relation between imitation and goal‐predictive gaze shifts in 12‐month‐old infants. In contrast to previous studies, which often presented the actions to be imitated on a computer monitor, we used a more naturalistic setting. In this setting, goal‐predictive gaze shifts were measured while a live model presented the actions at a realistic speed. Furthermore, we controlled for the infants' cognitive development.

Overall, the 12‐month‐old infants showed goal‐predictive gaze shifts for the observed action of putting the hat on the pig's head. Moreover, we found a relation between goal‐predictive gaze shifts and imitation; infants who imitated the action showed faster goal‐predictive gaze shifts than infants who did not imitate the action. However, goal‐predictive gaze shifts did not predict imitation when controlling for infants' cognitive development.

Looking at imitators and non‐imitators separately, both show goal‐predictive gaze shifts on the Goal AOI. Furthermore, both groups show goal‐predictive gaze shifts in the first trial. However, this does not necessarily mean that the infants did not learn actions during the demonstration phase, as they already predicted them in the first demonstration. The target state (the hat on the pig's head) was already familiar to the infants, as they knew it from the baseline phase and when the pig was first shown in the demonstration phase. In both cases, the pig was wearing the hat, so the infants already knew the target state and could predict it on the first trial. Furthermore, the pig is a high‐salience goal for the infants. Henrichs et al. (2012) found comparable goal‐predictive gaze shifts in 12‐month‐olds in the first trial with a high‐salience goal, whereas the infants tracked a low‐salience goal in the first trial; that is, their gaze did not reach the goal before the action was completed.

While previous research on goal‐predictive gaze shifts has primarily been based on screen‐based eye tracking, our study is among the first to investigate goal‐predictive gaze shifts in a more naturalistic context, with a live model presenting a specific goal‐directed action at real‐time speed. It adds to a growing body of literature addressing the need to investigate goal prediction in real‐world scenarios (Krol and Jellema 2022; Monroy et al. 2021). In line with Monroy et al. (2021), who investigated action prediction in free‐flowing real‐time infant‐parent interactions with six familiar toys in 9‐month‐olds, in the present study, 12‐month‐olds predicted the action goal in our setting. Similarly, Rosander and von Hofsten (2011) found that 10‐ to 11‐month‐olds show goal‐predictive gaze shifts in less than 1000 ms when the infants put a ball into a cylinder themselves and when they watched an experimenter perform this action live. Monroy and colleagues (2021), as well as Rosander and von Hofsten (2011), used two methods to investigate goal‐predictive gaze shifts in live settings. However, in both methods, the infants wore the devices directly on their bodies, whereby EOG, in particular, is very demanding for them. Our novel approach to study goal‐predictive gaze shifts in infants combines Monroy and colleagues' (2021) approach with the previously established approach of controlled, screen‐based laboratory paradigms (e.g., Falck‐Ytter et al. 2006s) by using live eye‐tracking in a controlled setting without directly affecting the infants. This allowed us to investigate infants' perception of specific actions presented at a realistic speed that approaches real‐life behavior.

In our setting, several potential distractors (e.g., the experimenter's face; presence of the caregiver) were present. Our results suggest that infants allocate their attention to the relevant target action at real‐time speed, even when these distractors are present. As such, the present findings indicate that in everyday life, infants are able to use real‐time interactions to build an understanding of social interactions and action goals of others, which constitutes the infants' learning environment.

Our second main finding is the relation between goal‐predictive gaze shifts and imitation. Infants who imitated Action Step 2 in the Pig task (“putting the hat back on”) showed faster goal‐predictive gaze shifts than infants who did not imitate this action. Previous studies found that infants' ability to perform a specific goal‐directed action correlates with understanding the action goal when another person performs this action (e.g., Cannon et al. 2012; Melzer et al. 2012). The present study shows that this relation between action perception and action production (see Prinz 1997) is also present in infants' imitation. This finding is in line with theories on infant imitation that highlight the role of infants' sensitivity to the agent's action goal, which assume that infants need to identify an agent's internal goal (Tomasello et al. 2005), or at least the external goal of an ongoing action (Gergely et al. 2002), to imitate this goal‐directed action. Recent studies further highlight that infants' imitation abilities may develop through their social experiences. For instance, Essler et al. (2023) present longitudinal evidence suggesting that being imitated by sensitive caregivers enhances infants' subsequent imitation abilities. This emphasizes the importance of contingent social interactions. This finding is consistent with the “cognitive gadget” theory (Heyes 2021), which posits that imitation is shaped by cultural learning mechanisms rather than innate cognitive instincts.

When interpreting the reported findings on the relation between imitation and goal‐predictive gaze shifts, it is important to consider the restrictive structure of the “Pig” task. Specifically, infants could only perform imitation of Action Step 2 (“putting the hat back on”) if they had succeeded in Action Step 1 (“removing the hat”). This sequential dependency may have limited the number of infants available for analysis of Step 2 and may partly explain the relatively low proportion of Step 2 imitators. Furthermore, the absence of differences in gaze arrival times between Step 1 imitators and non‐imitators, both of whom failed to perform Step 2, suggests that goal‐predictive gaze shifts alone were insufficient to enable the more complex two‐step imitation. Therefore, while the results provide evidence of a relation between goal‐predictive gaze and imitation, they also emphasize the importance of considering task structure and cumulative action demands when interpreting infants' imitation performance.

Goal‐predictive gaze shifts are related not only to imitation but also to other social‐cognitive processes. For instance, a correlation study reported a link between goal‐predictive gaze shifts and theory of mind in two‐year‐olds (Krogh‐Jespersen et al. 2015). The aforementioned study by Monroy et al. (2021) found a relation between goal‐predictive gaze shifts and child‐led joint attention, that is, children and parents attending to the same object, with the child looking at it first, in 9‐month‐olds. Moreover, another study found an association between goal‐predictive gaze shifts and the creation of an internal feedforward model in 6‐month‐olds (Gredebäck et al. 2018), that is, whether infants' goal prediction (looking at someone's mouth after seeing a moving spoon) is validated or proven incorrect (the spoon is directed elsewhere), resulting in a reaction of surprise. This further suggests that infants' goal prediction abilities are related to how they understand others' beliefs (Krogh‐Jespersen et al. 2015), learn from social interaction partners early in life (Monroy et al. 2021), and build an understanding of the behavior of others (Gredebäck et al. 2018).

Our third hypothesis was not confirmed. Although Step 2‐imitators showed faster goal‐predictive gaze shifts than non‐imitators, this difference was explained by infants' cognitive developmental status. This pattern of findings might be attributable to our study design, as we used only one imitation task of relatively high difficulty, which might have partially obscured the relation between goal prediction and imitation. Future studies might, therefore, use more than one task, with medium difficulty, to assess this relation. Furthermore, as our study is among the first to assess infants' cognitive development concerning the relation between goal‐predictive gaze shifts and imitation, future research should consider the role of infants' cognitive development in action prediction.

This finding highlights the importance of considering infants' general cognitive development when studying associations between social‐cognitive skills. Although faster goal‐predictive gaze shifts were observed in infants who imitated the target action, this relation became non‐significant once cognitive development was taken into account. This pattern is consistent with the results of other studies: for example, the relation between imitation and motor ability (Rogers et al. 2003) or attention following (Ingersoll and Meyer 2011) in children with autism was no longer evident once general cognitive development was taken into account. Together, these findings suggest that general cognitive development may be a common underlying factor that explains the links between different social‐cognitive abilities in infants, and that controlling for this factor is essential to accurately determine the specific contributions of individual skills.

One of the study's main strengths is its measurement of gaze‐arrival times during live demonstrations. However, this measurement also presents some challenges. Due to the live nature of the demonstration, the duration varies from trial to trial and from infant to infant. This means that the period during which an infant has the opportunity to anticipate varies from infant to infant. This variability makes it challenging to establish generally applicable time‐based criteria, for example with regard to inclusion criteria, or to define relevant time periods during which certain actions are of interest. The relation between gaze‐arrival time and the anticipation period further emphasizes the challenges of measuring gaze‐arrival times in live settings. These factors should be considered when designing new studies based on the present concept to avoid potential issues.

The present study has several limitations. First, less than 20% of the infants imitated the target action Action Step 2, and we analyzed only one item of the FIT 12 (out of five). Thus, future studies need to replicate these findings with a more extensive set of actions to assess action prediction and its association with action imitation more reliably. Another limitation of our methodology is that even if the infants looked at the experimenter's face, we could not include this behavior in our analysis. Faces, especially eyes, are potential sources of information for infants and, therefore, particularly interesting (Wagner et al. 2013). We could not analyze infants' gaze behavior toward the experimenter's face in our paradigm because the video recording focused on the presentation of the target action (see Figure 3). A future step would, therefore, be to include eye‐tracking across the whole scene into the paradigm to allow for conclusions about the impact of additional sources of information on infants' goal‐predictive gaze shifts. Another limitation of the study is the high dropout rates. Thirty‐three infants had to be excluded due to technical problems during calibration.

This study provides a novel approach to studying infants' perception of others' behavior by measuring goal‐predictive gaze shifts in a combined live eye‐tracking and imitation paradigm. The results suggest that similar to paradigms using computer monitors for stimulus presentation, infants also predict others' action goals in a more naturalistic setting that resembles the speed of activities in daily life. Moreover, the results highlight the importance of predicting and imitating others' behavior to understand social interactions. However, the question of whether goal‐predictive gaze shifts are one of the key processes involved in imitation remains unclear, as the results of the regression analysis emphasize the importance of cognitive development in infants in this association. Therefore, future studies should consider infants' cognitive developmental status in order to better understand the role of cognitive development in goal‐predictive gaze shifts and imitation. Overall, these findings emphasize a broader lesson: when investigating the interplay of social‐cognitive abilities in infancy, it is important to examine general cognitive development to avoid overstating domain‐specific associations.

## Author Contributions

**Franziska Sieber:** data curation, formal analysis, methodology, project administration, software, visualization, writing – original draft. **Jan Czarnomski:** data curation, investigation, methodology, software. **Moritz M. Daum:** conceptualization, supervision, writing – review and editing. **Norbert Zmyj:** conceptualization, funding acquisition, project administration, resources, supervision, validation, writing – review and editing.

## Ethics Statement

The present study was approved by the local Ethics Committee of the Faculty of Psychology at Ruhr University Bochum. The research was conducted in accordance with APA ethical standards in the treatment of the study sample.

## Conflicts of Interest

The authors declare no conflicts of interest.

## Supporting information


Supporting Information S1

## Data Availability

The data necessary to reproduce the analyses presented here are publicly accessible at https://osf.io/7rhzk/?view_only=8ac6570a6cf146819410c5803f4faa24. The analyses here are not preregistered.
