# Supplementary material for: The Relation Between Goal‐Predictive Gaze Behavior and Imitation—A Live Eye‐Tracking Study in 12‐Month‐Olds
Source: Infancy. 2025 Nov 4;30(6):e70050. doi: 10.1111/infa.70050 (PMC12586908; doi:10.1111/infa.70050)
Supplement: Supplementary file 1 — Supporting Information S1 [file INFA-30-0-s001.docx]

**Supplementary Materials**

**Definition criteria of the AOIs**

***Hat AOI***

The dynamic hat AOI must always cover the hat. In the first frame, its top edge is above the ball of the hand. The right edge is next to the hat. The left edge is next to the knuckle of the index finger and the bottom edge is under the hat. Consequently, the first dynamic AOI covers the hand and the hat. When the hand starts to move, the hat always has to be covered by the AOI and stays in its center. The last dynamic AOI is set next to the goal AOI and the pig AOI, when the hat enters the goal AOI, because the AOIs must not overlap.

***Goal AOI***

The static goal AOI covers the area on the head of the pig, where the hat is placed, the vertex of the pig’s head. The bottom edge is set directly above the eyes, so that it does not include the eyes. The left and right edge is set next to the respective ears. They are included in the AOI. The top edge covers a part of the demonstrators’ forearm. It has to be big enough that it covers the hat when it is placed on the vertex of the head of the pig.

***Pig AOI***

The static pig AOI is placed directly below the goal AOI. The top edge covers the pig's eyes. The bottom edge is placed under its feet. The left and right edges are placed so that the AOI covers the entire body of the pig, starting below the eyes.
